# Biostimulants for enhancing productivity, bioactive components, and the essential oils of garlic with the potential antifungal activity

**DOI:** 10.1186/s13568-024-01790-5

**Published:** 2024-11-27

**Authors:** Hanaa S. Hassan, Mostafa N. Feleafel, Mina S. R. Abd El-Lahot, Mervat EL-Hefny, Taghreed F. M. Abdel Rahman, Abeer A. Mohamed, Doaa Y. Abd-Elkader, R. M. Mahdy

**Affiliations:** 1 Department of Vegetable, Faculty of Agriculture (El-Shatby), Alexandria University, Alexandria, 21545 Egypt; 2 Department of Food Science and Technology, Faculty of Agriculture (El-Shatby), Alexandria University, Alexandria, 21545 Egypt; 3 Department of Floriculture, Ornamental Horticulture and Garden Design, Faculty of Agriculture (El-Shatby), Alexandria University, Alexandria, 21545 Egypt; 4 Department of Ornamental, Medicinal and Aromatic Plant Diseases, Plant Pathology Research Institute, Agricultural Research Center (ARC), Giza, 12619 Egypt; 5 Plant Pathology Institute, Agricultural Research Center (ARC), Alexandria, 21616 Egypt; 6 Horticulture Department, Faculty of Agriculture, Tanta University, Tanta, 31527 Egypt

**Keywords:** Essential oils, Foliar potassium fertilizer, Fungal inhibition, Garlic, Humic acid, Productivity

## Abstract

To feed the world’s growing population, the agriculture sector has recently had to strike a balance between reducing its detrimental effects on ecosystems and human health and boosting resource efficiency and production. In reality, pesticides and fertilizers are vital to agriculture and are useful instruments that farmers can employ to increase yield and guarantee steady productivity throughout the seasons under both favorable and unfavorable conditions. Therefore, in the present study, fertilizing with potassium citrate as a foliar spray and humic acid (HA) as a soil application allowed for the evaluation of vegetative growth parameters (plant height, number of leaves/plant), total phenolic content, total carbohydrate, antioxidant activity, the essential oil (EO) composition, and bulb yield of garlic (*Allium sativum* L.). These were carried out in two field experiments throughout the 2020–2021 and 2021–2022 growth seasons. A gas chromatography-mass spectroscopy (GC-MS) apparatus was performed to determine the chemical composition of the isolated EOs. The antifungal activity of the EOs was assessed against two fungi, *Fusarium proliferatum* and *Macrophomina phaseolina*, that cause geranium plants to wilt and decay. The findings indicated that applying HA at a rate of 2 g/L with potassium citrate at a rate of 5 or 10 mL/L produced garlic bulbs with the highest levels of productivity and diameter. The diverse treatments between HA with potassium citrate resulted in significant variations in the bioactive components, such as total phenol content, antioxidant activity, total carbohydrate, and sulfur content. The analysis of the EOs revealed the presence of dimethyl trisulfide, diallyl disulfide, methyl 2-propenyl trisulfide, allitridin, and methyl allyl disulfide and allyl tetrasulfide as main compounds. By gradually increasing the concentration of the garlic EO to 4000 µg/mL compared to the control, the inhibition percentage of fungal growth of *F. proliferatum* and *M. phaseolina* was increased. In conclusion, a high concentration of HA with potassium citrate (5 or 10 mL/L), may be suitable and highly appreciated as a fertilizer application to enhance the productivity and EOs content of garlic plants.

**Supplementary Information:**

The online version contains supplementary material available at 10.1186/s13568-024-01790-5.

## Introduction

Garlic (*Allium sativum* L. family Alliaceae) is one of the most important spice vegetables next to the onion throughout the world (Martins et al. 2016). According to the Food and Agriculture Organization Corporate Statistical Database, the total harvested area (16757 ha), total yield (207811 hg/ha), and total production (348230.7 tonnes) of garlic in Egypt were reported in 2021 (FAOSTAT 2022). The more than 5,000-year-old texts of the ancient Egyptians, Babylonians, and Indians all contain references to garlic (Palani et al. 2014; Rastogi et al. 2016). Garlic is a major bulbous vegetable crop grown worldwide especially in Egypt (Shafeek et al. 2015; Hassan et al. 2017; Petropoulos et al. 2018). There are many uses for garlic, however, it is most frequently used in cooking or as a spice or condiment (Mahala et al. 2022; Wang et al. 2022).

In terms of medicine, garlic has been shown to suppress the pathogenesis of cardiovascular disease, prevent cancer and other aging-related chronic disorders (Rahman 2003). Taking garlic supplements significantly lowers blood pressure (Chan et al. 2013). Garlic has long served as a valuable food and medicine (Jiku et al. 2020). Allicin, an organic sulfur molecule that gives garlic plants their unique odor, offers several physiological and therapeutic advantages (Arzanlou and Bohlooli 2010; Sayadi et al. 2020; Borlinghaus et al. 2021). Additionally, there is a significant concentration of non-volatile substances such as proteins, amides, phenolic compounds, antioxidants, certain minerals, and vitamin C (Lanzotti et al. 2014; Lu et al. 2011). Garlic has significant medical value and is used to treat some diseases due to its high mineral, vitamin, and allicin content (Gebreyohannes and Gebreyohannes 2013; Elosta et al. 2017).

A form of organic material called humic acid (HA) is mostly present in soil, water, and aged coal (He et al. 2022). HA is a dietary supplement with a high rate of nutrient utilization and is quickly absorbed (Pei et al. 2017). It has been demonstrated that applying HA increases the bioactivity and productivity of bulbous crops (Abdel-Razzak and El-Sharkawy 2013; Denre et al. 2014; Mohammed and Ibraheem 2020). Additionally, it was found that using HA fertilizer can increase agricultural yield by promoting plant growth, nutrient uptake, and metabolism (El-Nemr et al. 2012; Khan et al. 2013; Alkharpotly et al. 2017; Noroozisharaf and Kaviani 2018). Humic extracts applied topically to maize crops have boosted yield (fruit quantity and size) (García et al. 2016a, b). Liquid humus made from vermicompost of cattle dung has increased the crop yields in cucumber, rice, and broccoli (García et al. 2013, 2014). Additionally, liquid humus treatment to lettuce crops enhanced leaf area, total protein, and plant development (Hernandez et al. 2015).

Potassium (K) is a crucial component for vegetables, especially for root and bulb crops, where it plays a crucial role in quality, growth, and development. Numerous earlier studies examined how potassium affected the quantity and quality of bulb crops (Arisha et al. 2017; Zyada and Bardisi 2018; Metwaly et al. 2020; Wang et al. 2022). The total absorption ratio of N, P, and K in garlic plants is 1:0.3:0.7, therefore garlic plants need fertilizers with a high percentage of N and K, followed by P (Jiku et al. 2020). K fertilization became a significant role in Egypt’s production of vegetable crops. However, due to K fertilizer becoming a very expensive fertilizer, many farmers reduced the amount to the bare minimum or neglected to utilize it. K fertilizer is one of the key production techniques for the garlic crop. Simple, effective, and affordable methods, such as foliar spraying with additional K sources including potassium citrate, potassium silicate, and potassium thiosulfate, can be used to solve this issue (Behairy et al. 2015; El-Sayed et al. 2017; Shaban et al. 2018). Potassium citrate is a potassium salt of citric acid which is considered one of the important organic acids in the respiratory pathways in the cell (Ibrahim et al. 2015; El-Beltagi et al. 2017). Citric acid has a significant impact on how plants function metabolically, acting as a non-enzymatic antioxidant that chelates free radicals and shields plants from harm, extending the shelf life of plant cells, and enhancing growth characteristics (Okba et al. 2021). Garlic plant growth, yield, and storability are all enhanced by K treatment (EI-Morsy et al. 2004; Mahfooz et al. 2016). On average, combined treatments were more successful than solo ones at increasing garlic yield and bulb quality. These treatments included K levels and foliar sprays with mepiquat chloride (El-Sayed and El-Morsy 2012).

Different types of fertilizers have previously been used to increase the productivity and chemical composition of *A. sativum*. Leaves and bulbs of *A. sativum* under field conditions dramatically increased in cysteine, glutathione, and alliin content as sulfur fertilizer was applied, whereas N fertilization had no discernible effect (Bloem et al. 2010). The usage of ammonium sulfate resulted in an increase in garlic yield and its constituent yields, except clove weight, which did not increase (Zeinali and Moradi 2015). The foliar application of water-soluble fertilizers in Southern Gujarat (India) increased the growth, production, and quality characteristics of *A. sativum* var. Gujarat Garlic-3 (Mehta et al. 2017). Indole-3-butyric acid (IBA) reduced ion leakage and anthocyanin content in garlic, along with dramatically improved growth indices, bulb production, and allicin content (Bidmeshki et al. 2012). With the application of 60 kg of S/ha, plant height, number of leaves per plant, bulb diameter, number of cloves/bulb, weight of 10 cloves, average bulb weight, marketable yield, and total yield were greatly raised or improved (Babaleshwar et al. 2017).

Alliin or S-allyl-cysteine sulfoxide is the most common taste precursor, and thiosulfinates are the major chemicals responsible for the flavor of garlic (Horníčková et al. 2010). Enzymatic cleavage of alliin to allicin and its derivatives releases the distinctive smell of garlic (Bloem et al. 2010). Diallyl sulfide, diallyl disulfide, diallyl trisulfide, and ajoene are examples of oil-soluble chemicals that are more potent prospective cancer treatments than water-soluble ones (Choromanska et al. 2020). Different extraction techniques revealed that allyl polysulfides compounds, such as diallyl sulfide, diallyl disulfide, diallyl trisulfide, allyl methyl disulfide, and allyl methyl trisulfide, predominated in the essential oils (EOs) obtained from the bulbs (Satyal et al. 2017).

Several fungal plant diseases can be controlled by garlic EOs as an antimicrobial agent (Li et al. 2014; Gwinn 2018; Mugao et al. 2020). Garlic juice can stop *Phytophthora infestans* and *Pseudoperonospora cubensis* from infecting cucumbers (Portz et al. 2008). Applying crude extracts and EOs from garlic helps prevent the early and late blight fungal diseases in tomatoes caused by *Alternaria solani* and *P. infestans*, respectively (Mugao et al. 2020). At concentrations as low as 20%, the postharvest pathogens *Botrytis cinerea*, *Penicillium expansum*, and *Neofabraea alba* were inhibited by the volatile vapor application of garlic, and *B. cinerea* and *P. expansum* were inhibited by aqueous and ethanol dilutions on media supplemented with garlic extract (Daniel et al. 2015).

In Egypt, root rot and wilt caused by *Macrophomina phaseolina* and *Fusarium proliferatum* are the two most common geranium diseases (Adolf 2016; Ghazi et al. 2018; Mishra et al. 2022; Abdel-Rahman et al. 2023). The most significant medicinal aromatic crop in Egypt and the entire world is geranium (*Pelargonium graveolens* L.), a member of the Geraniaceae family (Lothe and Verma 2023b). Geranium is frequently grown as an ornamental plant due to its beautiful attributes. Geranium EOs are widely used in medicine, pharmaceutical sector, agricultural industry, gastronomy, cosmetics, and pesticides (Lothe and Verma 2023a). Geraniums are susceptible to root-rot and wilt illnesses caused by fungal pathogens that are primarily soil-borne and can be spread by terminal cuttings. This has a significant negative impact on the output and quality of geranium oil in Egypt and around the world (Ghazi et al. 2018). In Egypt, the prevalence of these illnesses is always rising, and the signs of wilt, root rot, and Geranium yellowing are worse every year (Abdel-Rahman et al. 2023). This can be because there are no efficient disease control measures in place in the field or nursery.

Given the significance of humic acid and potassium availability for the cultivation of garlic crops, the objective of this study was to assess the effects of various humic acid and potassium citrate treatments on productivity, bioactivity of garlic bulbs, and the essential oil percentages. Additionally, it was determined that using garlic essential oils was a risk-free strategy to control the spread of the diseases that cause geranium plants to wilt and decay, *Macrophomina phaseolina*, and *Fusarium proliferatum*.

## Materials and methods

### Experimental site

Two field experiments on a garlic vegetable crop were carried out in the Faculty of Agriculture, Alexandria University, Egypt (31.200° N and 29.9187° E) at the Experimental Station Farm between September and April in the years 2020/2021 and 2021/2022. Before initiating the two experiments, soil samples were extracted from the upper 25 cm layer for chemical analyses according to the standard procedures (Sparks et al. 2020). The soil type is clay with pH: 8.2- 8.0, EC: 2.3–2.1 dS/m, total N: 0.16, 0.18%, P: 30, 28 ppm, K: 0.33, 0.36 m eq/L, and Ca: 1.9, 2.0 m eq/L, in the first and second season, respectively.

### Experimental design and management

Clone Sids-40 garlic treatments and crop management was adopted in both study experiments. It is known by its big clove size, easy peer, whose mature cloves have bright white skin with purple vertical stripes. Cloves of garlic were manually sown in half of September in both seasons and were planted upright with the apical tip on both sides of the rows 4 m in length and 60 cm width apart with a spacing of 10 cm between cloves. The proposed study was arranged in a randomized complete block design with three replications. Each replicate included nine treatments, which were the combinations of three injections of humic acid (HA) on soil and three rates of potassium citrate as a foliar application.

The rates of HA (0, 1, and 2 g/L (w:v) were randomly arranged in the main plots, while potassium citrate rates (0, 5, and 10 mL/L (v:v) were, randomly, distributed in the sub-plots. Each plot consisted of 12 ridges having an area of 28.8 m^2^. HA (potassium humate, 85% humate, and 15% potassium were mixed with water and each plant took about 0.2 L) rates were added three times as a drench to the plant root area; 45, 60, and 75 days after planting. While, potassium citrate levels were applied three times as foliar application after 120, 135, and 150 days from the planting date. The plants were nitrogen fertilizer at the rate of (200 kg/ha) which was added after 30, 60, and 90 days of planting. While phosphorus fertilizer was applied two times during the preparation of the soil and 30 days after planting (Hassan et al. 2017). The levels of HA and potassium citrate treatments are shown in Table S1.

### Vegetative growth characteristics

A sample of five plants from each experimental unit was taken after planting for vegetative growth data. Plant height and number of leaves/plant.

### Evaluation of the productivity

At harvesting time in April and after curing for 21 days in a clean, shaded, well-ventilated, and dry room, the total yield weighed for each treatment after curing was measured and attributed to the hectare (ton). Ten bulbs were taken randomly from each experimental unit to measure the bulb diameter (cm), nick diameter (cm), and bulb ratio, which was measured as follows: Bulb ratio (%) = neck diameter (cm)/bulb diameter (cm) × 100.

### Bioactive components of bulbs

To determine the total phenolic content, the bulbs’ samples were extracted by methanol containing 0.1% HCl. The total phenolic content expressed as gallic acid equivalent (GAE)/100 g of fresh weight (FW) was measured at 750 nm by Optizin UV–Vis spectrophotometer model ne (Thermo Electron Corporation, Waltham, MA, USA) described by A.O.A.C. (2000).

To determine the antioxidant activity, a sample of about 10 g from a fresh garlic bulb was soaked in 50 mL of ethanol (80%) for 1 week at room temperature and then filtered through Whatman paper No 1. The antioxidant activity was assessed by evaluating the free-radical-scavenging activity of the 2,2-diphenyl-1-picryl-hydrazyl (DPPH) radical according to a modified method as described previously (Hwang and Thi 2014) using Optizin UV–Vis spectrophotometer model (Thermo Electron Corporation, Waltham, MA, USA). The radical-scavenging activity was calculated as a percentage of DPPH discoloration using the following equation: scavenging activity (%) = (A_Control_– A_Sample_)/A_Control_) ×100, where: A_Sample_ is the absorbance of the tested sample and A_Control_ is the absorbance of the control (DPPH solution).

Total carbohydrate in bulbs (%) was measured in dry matter of bulb (Ahmed 1994). Sulphur (mg/g.d.w) was determined as Sulphur dioxide using the iodine titration method (Ranganna 1986).

### Extraction of the essential oils by hydrodistillation method

*Allium sativum* bulbs collected after harvesting from the treated plants with several combinations treatments of HA and potassium citrate were chopped into small pieces and were subjected to a distillation method using a Clevenger apparatus for 3 h (Abd El-Kareem et al. 2024; Fikry et al. 2019). The obtained pale yellow essential oils (EOs) were passed through sodium chloride to remove the remaining water. The percentage of the EOs was calculated based on the FW of bulbs. The collected EOs were stored under refrigeration (− 4 °C) until analysis (Satyal et al. 2017).

### Gas chromatography-mass spectrometry (GC-MS) analysis

The chemical composition of the EOs was performed using a GC-TSQ mass spectrometer (Thermo Scientific, Austin, TX, USA) with a direct capillary column TG–5MS (30 m x 0.25 mm x 0.25 μm film thickness). The column oven temperature was initially held at 60 °C and then increased by 5 °C/min to 250 °C withheld for 2 min then increased to 280 at 25 °C/min. The injector temperature was kept at 270 °C. Helium was used as a carrier gas at a constant flow rate of 1 mL/min. The solvent delay was 4 min and diluted samples of 1 µL were injected automatically using Autosampler AS3000 coupled with GC in the split mode. EI mass spectra were collected at 70 eV ionization voltages over the range of m/z 50–650 in full scan mode. The ion source and transfer line were set at 200 °C and 280 °C respectively. The components were identified by comparison of their mass spectra with those of WILEY 09 and NIST14 mass spectral database (Abd El-Kareem et al. 2016).

### Antifungal activity assays of garlic essential oil

*Macrophomina phaseolina* (AUMC15578) and *Fusarium proliferatum* (AUMC15577) were isolated from naturally infected geranium (*Pelargonium graveolens* L.) (Abdel-Rahman et al. 2023). The culture of two fungi isolates was assayed on the agar dilution method was carried out through the combination of different required concentrations of the EO into the potato dextrose agar medium (PDA). A single 5-mm culture disk containing the isolates of *M. phaseolina* and *F. proliferatum* was taken from actively growing cultures and placed in the Petri dishes in the middle (Shakam et al. 2022; El-Hefny et al. 2023). Briefly, the EO was prepared at concentrations of 4000, 1000, 250, 60, and 15 µg/mL by diluted in 0.01% dimethyl sulfoxide (DMSO) with the addition of a few drops of Tween-80 (0.01%) as an emulsifier to dilute the EOs (Abd El-Kareem et al. 2024; Mohamed et al. 2020a, b), were prepared and compared with referenced fungicide, methyl benzimidazol-2-ylcarbamate (Occidor^®^ 50% SC) (Chimac Agriphar S.A., Belgium) was tested at their recommended dosage (2 g/L) for antifungal activity by the poisoned food technique (Gupta et al. 2015). By the fungal discs in the center, the controls were solely kept in a PDA medium. Based on the measurement of linear growth of *M. phaseolina* and *F. proliferatum* on the agar surface surrounding the well, the antifungal activity was evaluated. The plates were incubated for a week at 28 °C, and the minimum inhibitory concentrations (MICs) were those at which the growth of the fungus was inhibited (Kottearachchi et al. 2012). Inhibition percentage of fungal growth % (IPFG) of the tested *M. phaseolina* and *F. proliferatum* were calculated with the following formula (Hassan et al. 2021; Salem et al. 2021): (IPFG) (%) = [DC-DT/DC] × 100, where DC and DT are the average diameters (mm) of fungal colonies under the control and experimental treatments, respectively.

### Statistical analysis

All data were analyzed by the CoStat software version 6.303 (CoHort Software 798 Lighthouse Ave. PMB 320, Monterrey, CA, 93940, USA) package through a two-way analysis of variance (ANOVA) in a split-plot design. The least significant difference (LSD) test (*p* < 0.05) was used to separate the means (Steel and Torrie 1980). In a split-plot design, the subplot (potassium citrate) is given more weight than the main plot (humic acid), whereas in a factorial experiment, all components are given equal weight. The main plot is more inaccurate than the subplot. Therefore, we need more precise data for the potassium citrate than for the humic acid we were aiming for.

## Results

### Impact of applied treatments on vegetative growth traits

**Fig. 1:**
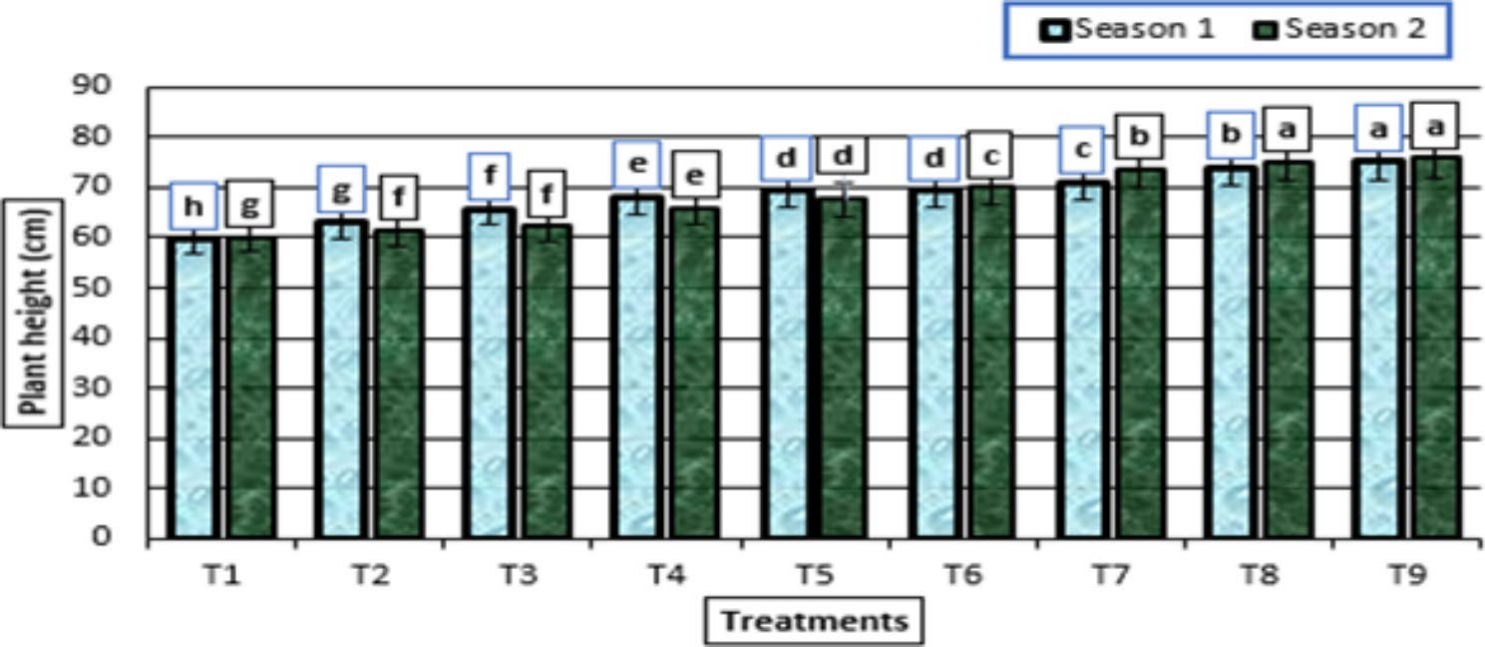
Plant height (cm) (means ± S.E) of garlic as affected by different levels of potassium citrate and HA in season 1 and season 2. Letters in Figure indicated that, means ± S.E of treatments with the same letter/s were not significantly different according to LSD at 0.05 level of probability. T1: untreated plants; T2: HA (0 g/L) + potassium citrate (5 mL/L); T3: HA (0 g/L) + potassium citrate (10 mL/L); T4: HA (1 g/L) + potassium citrate (0 mL/L); T5: HA (1 g/L) + potassium citrate (5 mL/L); T6: HA (1 g/L) + potassium citrate (10 mL/L); T7: HA (2 g/L) + potassium citrate (0 mL/L); T8: HA (2 g/L) + potassium citrate (5 mL/L); T9: HA (2 g/L) + potassium citrate (10 mL/L)

**Fig. 2:**
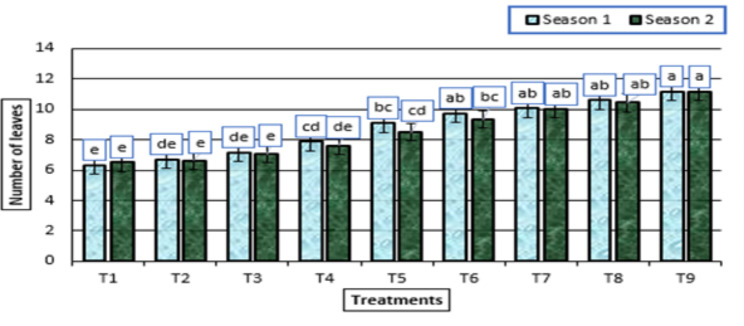
Number of leaves (means ± S.E) of garlic as affected by different levels of potassium citrate and humic acid in season 1 and season 2. Letters in Figure indicated that, means ± S.E of treatments with the same letter/s were not significantly different according to LSD at 0.05 level of probability. Legends for T1 to T9, are shown in Fig. 1

The use of potassium fertilizer and humic acid (HA) boosted plant height and leaf number in the garlic plant compared to the control plant without potassium citrate fertilizer and HA (Figs. 1 and 2). The most successful treatment, which enhanced the plant’s height and number of leaves in both growth seasons, was the administration of HA (2 g/L) + potassium citrate (10 mL/L) to the garlic plants (Figs. 1 and 2).

### Effect of treatment application on garlic productivity

**Fig. 3:**
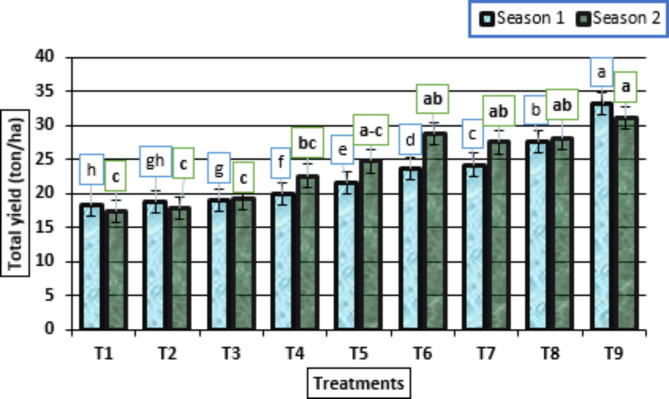
Total yield (ton/ha) (means ± S.E) of garlic as affected by different levels of potassium citrate and humic acid in season 1 and season 2. Letters in Figure indicated that, means ± S.E of treatments with the same letter/s were not significantly different according to LSD at 0.05 level of probability. Legends for T1 to T9, are shown in Fig. 1

As shown in Fig. 3, the application of the treatment HA (2 g/L) + potassium citrate (10 mL/L), had the highest positive effects, in the two growing seasons of the total yield (ton/ha) compared with the other treatments. On the other hand, the total yield of garlic (ton/ha) from untreated plants to treatment plants with HA (1 g/L) + potassium citrate (5 mL/L) was lower than that of the treated plants with HA (1 g/L) + potassium citrate (10 mL/L) to HA (2 g/L) + potassium citrate (10 mL/L). Moreover, the total yield increased gradually as the HA and potassium citrate application rate increased.

**Fig. 4:**
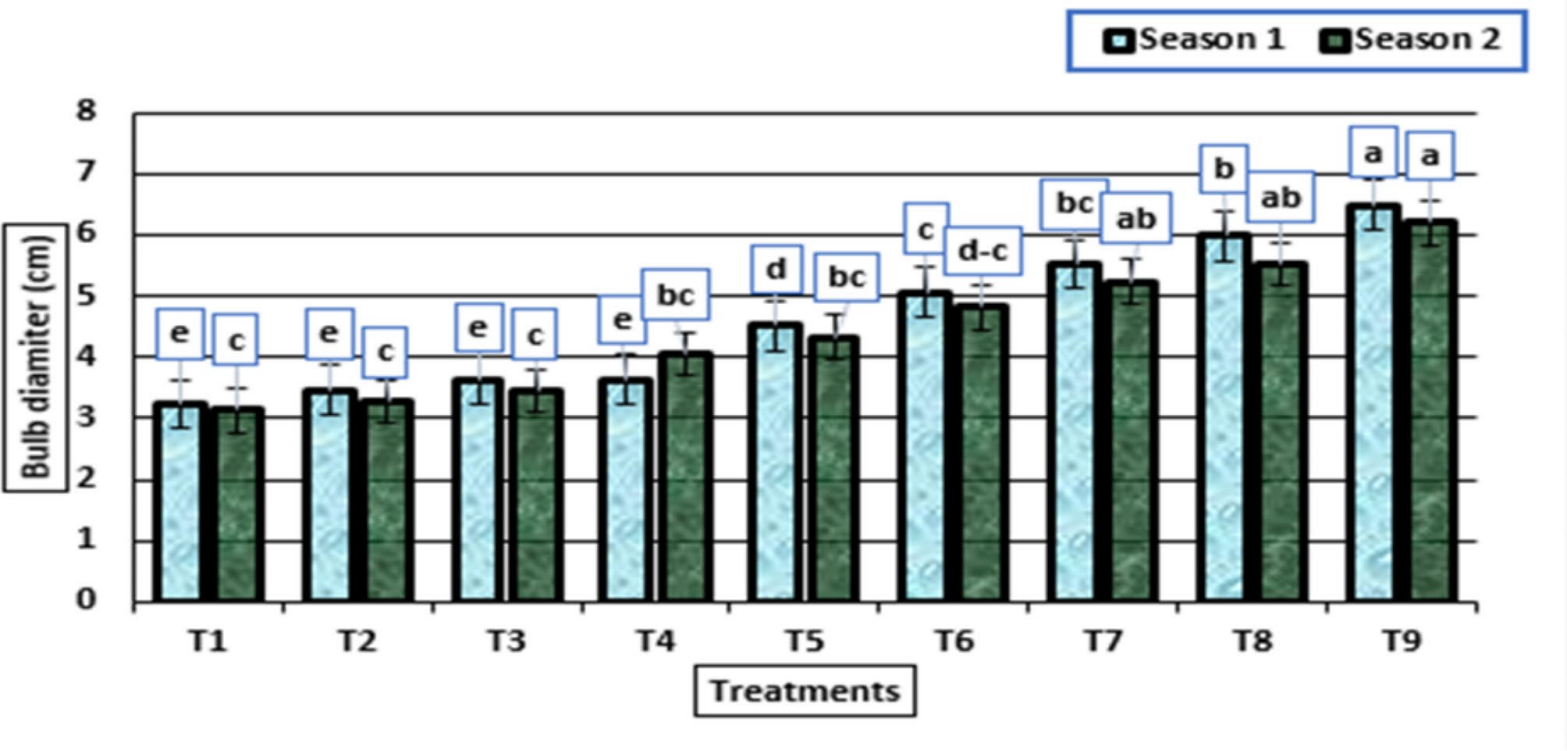
Bulb diameter (cm) (means ± S.E) of garlic as affected by different levels of potassium citrate and humic acid in season 1 and season 2. Letters in Figure indicated that, means ± S.E of treatments with the same letter/s were not significantly different according to LSD at 0.05 level of probability. Legends for T1 to T9, are shown in Fig. 1

**Fig. 5:**
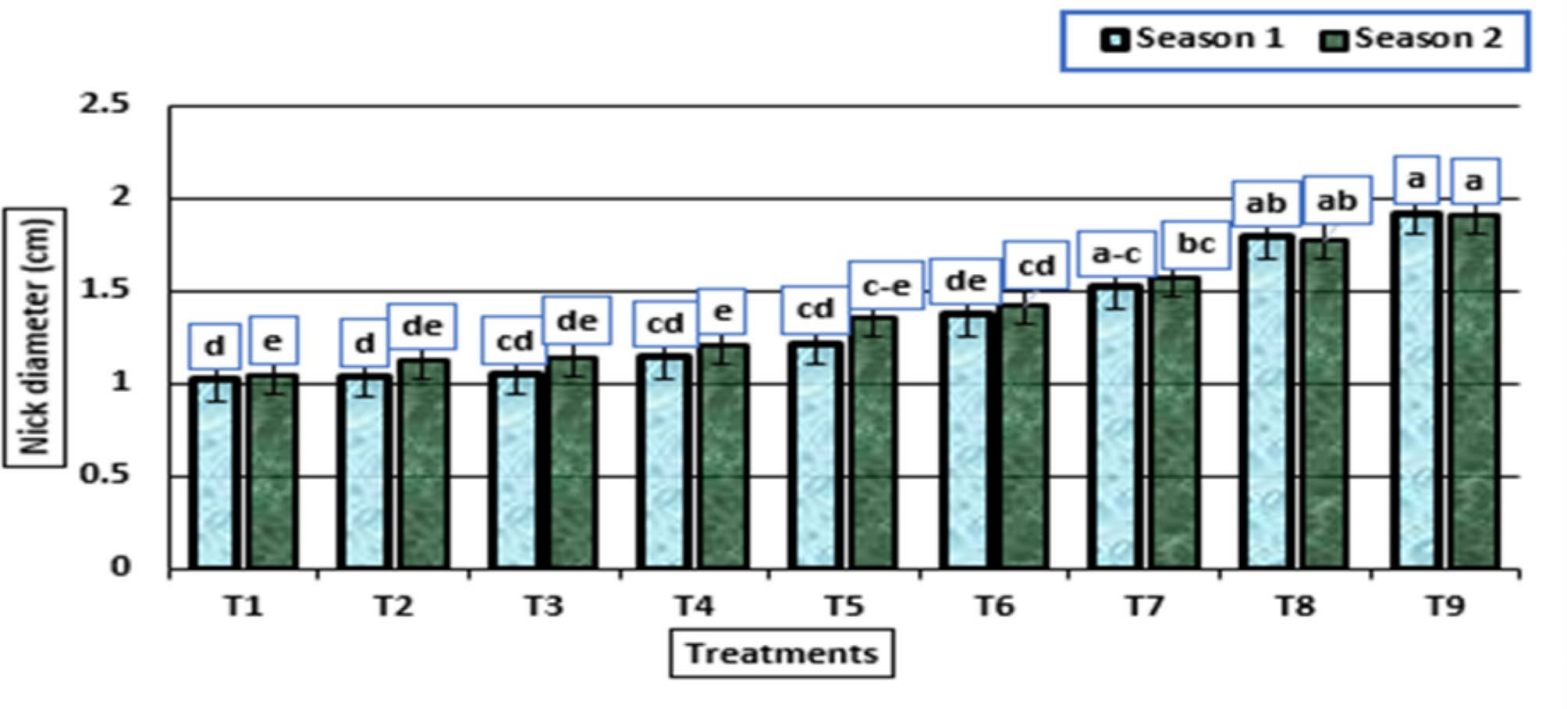
Nick diameter (cm) (means ± S.E) of garlic as affected by different levels of potassium citrate and humic acid in season 1 and season 2. Letters in Figure indicated that, means ± S.E of treatments with the same letter/s were not significantly different according to LSD at 0.05 level of probability. Legends for T1 to T9, are shown in Fig. 1

As shown in Figs. 4 and 5, the bulb and neck diameter were significantly affected by the different treatments in both seasons. There were no significant differences among the treatments: untreated plants, treated with HA (0 g/L) + potassium citrate (5 mL/L) and with HA (0 g/L) + potassium citrate (10 mL/L), while the diameters increased for both characteristics, and there was a significant increase from the treated plants with HA (1 g/L) + potassium citrate (0 mL/L) to treated plants with HA (2 g/L) + potassium citrate (10 mL/L), as a result of increasing the concentrations of HA and potassium citrate used. In addition, the largest bulb and nick diameter were obtained by the treatment HA (2 g/L) + potassium citrate (10 mL/L) in both growing seasons.

**Fig. 6:**
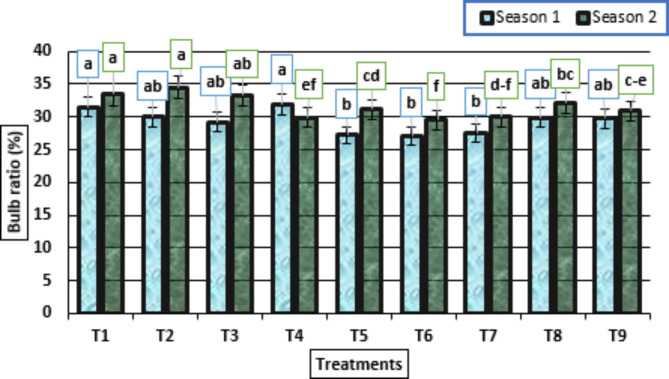
Bulb ratio (%) (means ± S.E) of garlic as affected by different levels of potassium citrate and humic acid in season 1 and season 2. Letters in Figure indicated that, means ± S.E of treatments with the same letter/s were not significantly different according to LSD at 0.05 level of probability. Legends for T1 to T9, are shown in Fig. 1

The garlic plants treated with different treatments of HA with potassium citrate showed that the untreated plants had a highly significant increase in the studied bulb ratio (%) trait in both seasons (Fig. 6). Generally, the treatments were ordered as untreated plants, treated plants with HA (0 g/L) + potassium citrate (5 mL/L), and with HA (0 g/L) + potassium citrate (10 mL/L) in descending order for their positive effects on bulb ratio (%) trait, in both growing seasons (Fig. 6).

### Effect of the application of the treatments on the bulb bioactive components

Under the field conditions, there was a significant difference in bulb bioactive components in response to the nine treatments (Table 1). Total phenol content (TPC) in the methanol containing 0.1% HCl extracts from the bulbs from untreated plants (control) treatment (~ 28 mg GAE/100 g f.w.) was substantially lower (*p* < 0.05) than the value from the treated plants with HA (2 g/L) + potassium citrate (5 mL/L) (~ 67 mg GAE/100 g f.w.). However, despite this difference, TPC in bulbs was significantly increased when HA and potassium citrate were applied to the garlic plant.

**Table 1:** The phenolic content, antioxidant activity, total carbohydrate, and sulfur content of garlic cloves as affected by different levels of potassium citrate and humic acid

Treatments	Total phenol content (mg GAE/100 g f.w.)		Antioxidant activity (% capacity)		Total carbohydrate (%)		Sulfur (mg/g F.w)	
	Season 1	Season 2	Season 1	Season 2	Season 1	Season 2	Season 1	Season 2
T1	27.93 ± 1.10f^*^	28.91 ± 2.89c	19.44 ± 0.43d	19.34 ± 1.39f	25.64 ± 0.1e	24.93 ± 1.2g	3.02 ± 1.4e	2.88 ± 2.1e
T2	55.23 ± 1.54e	55.58 ± 2.65b	22.63 ± 0.43c	22.49 ± 1.09de	26.31 ± 0.2de	25.21 ± 1.1f	3.27 ± 1.3e	3.01 ± 2.2e
T3	58.91 ± 0.58c	58.85 ± 1.21b	26.88 ± 1.31b	25.71 ± 1.18b	27.43 ± 0.1d	26.44 ± 0.6e	4.14 ± 1.4de	4.04 ± 1.9de
T4	27.78 ± 0.44f	29.40 ± 2.02c	19.55 ± 0.43d	19.62 ± 1.28f	30.36 ± 0.1c	29.87 ± 0.5d	4.45 ± 1.1c-e	4.32 ± 1.8de
T5	59.36 ± 0.48bc	59.65 ± 2.31b	22.80 ± 1.51c	23.87 ± 1.28c	31.44 ± 0.2b	31.42 ± 0.2c	4.61 ± 1.1c-e	4.41 ± 2.3c-e
T6	56.86 ± 2.28d	58.17 ± 1.72b	27.57 ± 0.46b	27.78 ± 1.12b	33.21 ± 0.3b	33.73 ± 0.4b	5.53 ± 0.1b-d	5.25 ± 2.4b-d
T7	57.59 ± 0.83d	57.65 ± 2.03b	23.27 ± 0.43c	23.44 ± 1.20c	35.20 ± 0.3b	34.44 ± 1.2ab	6.14 ± 1.5a-c	5.89 ± 2.2a-c
T8	66.99 ± 2.82a	67.78 ± 1.21a	32.48 ± 0.24a	32.41 ± 0.84a	36.44 ± 0.4a	35.46 ± 1.3a	6.77 ± 1.6ab	6.59 ± 1.9ab
T9	60.20 ± 1.21b	59.91 ± 1.19b	31.38 ± 0.26a	31.45 ± 1.18a	36.86 ± 0.3a	36.95 ± 0.7a	7.04 ± 1.5a	7.12 ± 1.3a

*: Means with the same letter/s within the same column are not significantly different according to LSD at 0.05 probability level. Legends for T1 to T9, are shown in Fig. 1

There was a significant variation in the antioxidant activity (AA%) by ethanol extracts from garlic bulbs due to the effect of different treatments (Table 1). A significant variation in AA% by garlic bulb extracts was observed due to the application of treatments HA (2 g/L) + potassium citrate (5 mL/L) and HA (2 g/L) + potassium citrate (10 mL/L). The highest AA (32.48%) was observed in ethanol extract from the plants treated with HA (2 g/L) + potassium citrate (5 mL/L), and the lowest AA (19.34%) was observed in the untreated plants in both growing seasons. Additionally, it is clear from the data in Table 1 that application of garlic plant with HA (2 g/L) + potassium citrate (5 mL/L) and HA (2 g/L) + potassium citrate (10 mL/L) resulted in a significant increase in total carbohydrate (%) of bulbs compared with other treatments.

The sulfur content was positively increased with the increases in the HA concentration combined with potassium citrate applications of the garlic plant, which significantly increased in both seasons as plants treated with HA (2 g/L) + potassium citrate (5 mL/L) (6.77, 6.59 mg/g F.w.) and HA (2 g/L) + potassium citrate (10 mL/L) (7.04, 7.12 mg/g F.w.).

### The essential oil percentages and chemical constituents by GC-MS

**Fig. 7:**
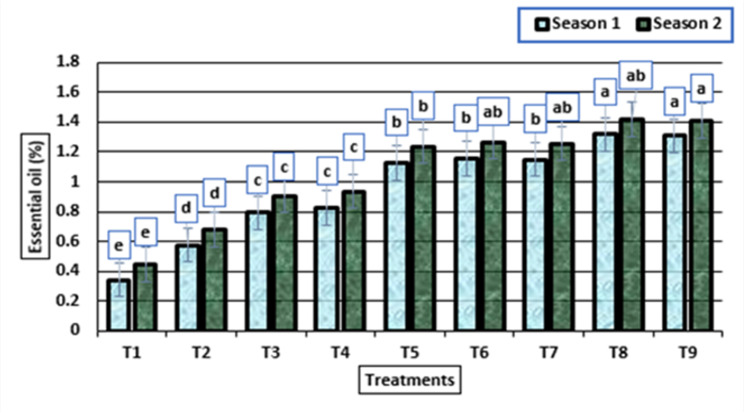
Essential oil (%) (means ± S.E) of garlic bulbs as affected by different levels of potassium citrate and humic acid in season 1 and season 2. Letters in Figure indicated that, means ± S.E of treatments with the same letter/s were not significantly different according to LSD at 0.05 level of probability. Legends for T1 to T9, are shown in Fig. 1

In the first season, the percentages of pale yellow EOs ranged from 0.34 (untreated plants) to 1.31% (plants treated with HA 2 g/L + potassium citrate 5 mL/L), while in the second season, they ranged from 0.44 to 1.41% with the same treatments. Additionally, the treatment HA 2 g/L + potassium citrate 10 mL/L displayed EO percentage values of 1.307 and 1.407%, in the first and second seasons, respectively (Fig. 7).

Table 2 and Fig. S1 display the chemical components of garlic EOs as they are impacted by various treatments. The concentration of dimethyl trisulfide ranged from 1.57 to 3.72%, with the highest amount (3.72%) discovered in the EO from plants treated with HA (0 g/L) + potassium citrate (10 mL/L), followed by plants treated with HA (1 g/L) + potassium citrate (0 mL/L) (3.64%), and plants treated with HA (1 g/L) + potassium citrate (5 mL/L) (3.49%).

**Table 2:** The chemical compounds identified in garlic essential oils as affected by several treatments

Compound name	T1	T2	T3	T4	T5	T6	T7	T8	T9
Dimethyl trisulfide	3.22* (948)	0.91 (891)	3.72 (958)	3.64 (943)	3.49 (939)	3.32 (941)	ND	1.57 (945)	ND
Diallyl disulfide	13.86 (918)	10.01 (918)	14.31 (923)	14.17 (923)	14.96 (923)	14.66 (920)	5.93 (907)	11.86 (920)	2.95 (902)
(E)-1-Allyl-2-(prop-1-en-1-yl)disulfane	0.94 (775)	ND	0.81 (765)	0.78 (816)		0.79 (757)	ND	0.67 (761)	ND
(Z)-1-Allyl-2-(prop-1-en-1-yl)disulfane	0.65 (844)	ND	ND	0.50 (836)	ND	ND	ND	0.47 (818)	ND
Dipropyl disulfide	0.98 (910)	0.65 (848)	1.00 (896)	0.96 (891)	0.99 (878)	1.01 (889)	0.43 (759)	0.78 (877)	ND
Methyl 2-propenyl trisulfide	15.31 (976)	12.48 (914)	15.07 (976)	14.42 (917)	15.85 (929)	15.30 (923)	11.72 (882)	13.65 (916)	11.91 (903)
4-Methyl-1,2,3-trithiolane	ND	1.44 (869)	1.08 (825)	0.96 (867)	ND	1.14 (858)	1.49 (867)	ND	1.37 (872)
1,4-Dimethyltetrasulfane	1.67 (923)	1.93 (912)	2.12 (919)	1.96 (923)	2.26 (931)	2.12 (925)	2.13 (926)	1.96 (933)	1.89 (927)
9-Thiabicyclo[3.3.1]non-7-en-2-ol	ND	ND	ND	0.39 (727)	ND	ND	0.58 (737)	ND	ND
Trisulfide, di-2-propenyl (Allyl trisulfide) or Allitridin	28.23 (908)	36.02 (915)	30.87 (904)	30.57 (919)	33.96 (912)	31.98 (906)	40.06 (914)	32.01 (906)	37.69 (907)
1-Allyl-3-propyltrisulfane	1.86 (898)	2.28 (881)	2.16 (911)	1.98 (912)	2.30 (901)	2.20 (907)	2.54 (892)	2.03 (893)	2.33 (885)
Trisulfide, dipropyl	ND	0.93 (804)	1.00 (866)	0.80 (799)	ND	1.04 (814)	1.14 (787)	0.97 (797)	1.15 (843)
5-Methyl-1,2,3,4-tetrathiane	1.20 (864)	2.44 (884)	1.99 (885)	1.81 (880)	2.26 (891)	2.05 (898)	2.56 (887)	1.65 (885)	2.54 (885)
Methyl allyl disulfide	10.03 (828)	10.06 (820)	8.60 (831)	8.04 (775)	8.61 (778)	7.85 (813)	8.49 (793)	9.91 (827)	9.93 (818)
1,4-Ethanonaphthalene-2-carboxylic acid, 1,4-dihydro-, methyl ester	ND	ND	ND	0.34 (728)	ND	ND	0.42 (706)	ND	0.47 (601)
1-(1-(Methylthio)propyl)-2-propyldisulfane	ND	ND	ND	0.34 (629)	ND	ND	0.50 (697)	ND	0.51 (797)
5-Hexyldihydro-2(3 H)-furanthione	1.03 (623)	1.26 (622)	1.08 (627)	1.00 (632)	1.17 (633)	1.08 (620)	1.34 (632)	1.20 (622)	1.39 (638)
1-[2-Deoxy-á-d-erythro-pentofuranosyl]pyrrole-2,4-bisthiocarboxamide	0.85 (629)	1.01 (633)	0.88 (621)	0.83 (637)	0.97 (645)	0.91 (631)	1.10 (659)	1.00 (622)	1.13 (617)
3-Ethoxycarbonylmethyl-2-imino-2,3-dihydrothiazole	ND	ND	ND	0.41 (852)	ND	ND	0.54 (678)	ND	0.57 (699)
Allyl tetrasulfide	13.48 (905)	12.64 (907)	10.55 (897)	9.65 (898)	10.84 (896)	9.85 (906)	10.90 (908)	13.06 (904)	13.01 (898)
1-Allyl-2-isopropyldisulfane	1.21 (799)	1.09 (787)	0.98 (793)	0.83 (792)	ND	0.97 (783)	1.06 (766)	1.20 (786)	1.24 776
Disulfide, methyl 1-(1-propenylthio)propyl	ND	ND	ND	0.62 (804)	ND	ND	0.79 (777)	0.74 (790)	0.85 (793)
Benzoic acid, 4-(1,1-dimethylethoxy)-	ND	ND	ND	ND	ND	ND	ND	ND	0.64 (683)
Propane, 1,1’-thiobis[3-(methylthio)-	ND	ND	ND	0.31 (714)	ND	ND	ND	ND	0.46 (681)
5-Oxohexanethioic acid, S-t-butylester	0.99 (640)	1.22 (664)	0.98 (637)	0.91 (636)	1.09 (647)	1.00 (624)	1.18 (665)	1.11 (699)	1.25 (710)
1,5-Dithiaspiro[5.6]dodecan-7-ol	ND	ND	ND	ND	ND	ND	0.57 (716)	ND	0.58 (701)
1-Allyl-3-(2-(allylthio)propyl)trisulfane	1.16 (874)	1.18 (858)	0.93 (881)	0.88 (868)	ND	0.93 (871)	1.08 (841)	1.15 (877)	1.19 (850)
4,4,5,6-Tetramethyl-3,4-dihydro-2 H-1,3-thiazin-2-thione	ND	ND	0.71 (646)	0.66 (688)	ND	0.70 (604)	0.84 (637)	0.85 (628)	0.92 (638)
2-[Bis(methylsulfanyl)methylene]cyclohexanone	1.40 (622)	1.51 (637)	1.17 (629)	1.06 (624)	1.27 (655)	1.11 (617)	1.36 (629)	1.41 (612)	1.41 (607)
8-(Benzoyloxy)quinoline	1.06 (613)	ND	ND	0.53 (750)	ND	ND	0.60 (766)	0.75 (709)	ND
1-Allyl-3-(2-(allyldisulfanyl)propyl)trisulfane	0.88 (911)	ND	ND	ND	ND	ND	ND	ND	ND
10-Octadecenoic acid, methyl ester	ND	ND	ND	ND	ND	ND	ND	ND	0.50 (824)
4-Vinylbenzylchloride	ND	ND	ND	ND	ND	ND	ND	ND	0.93 (688)

*Values are compound percentages (match factor). ND: not detected

Diallyl disulfide was found in the range of 2.95–14.96%, where the highest amount (14.96%) was found in EO from garlic plants treated with HA (1 g/L) + potassium citrate (5 mL/L) followed by HA (1 g/L) + potassium citrate (10 mL/L) (14.66%), HA (0 g/L) + potassium citrate (10 mL/L) (14.31%), and HA (1 g/L) + potassium citrate (0 mL/L) (14.17%) and the lowest amount observed with the treatment of HA (2 g/L) + potassium citrate (10 mL/L) (2.95%).

Methyl 2-propenyl trisulfide was found in the range of 11.72–15.85%, where the highest amount was reported in the EO of garlic plants treated with HA (1 g/L) + potassium citrate (5 mL/L) (15.85%) followed by untreated plants (15.31%), treated plants with HA (1 g/L) + potassium citrate (10 mL/L) (15.30%) and HA (0 g/L) + potassium citrate (10 mL/L) (15.07%), while the lowest amount was found in the oil of plants treated with HA (2 g/L) + potassium citrate (0 mL/L) (11.72%).

Trisulfide, di-2-propenyl (allyl trisulfide or allitridin) was found in the high percentages and ranged from 28.23 to 40.06% in the EO from plants treated with untreated plants and treated with HA (2 g/L) + potassium citrate (0 mL/L), respectively. Furthermore, the EO from the treated plants with HA (0 g/L) + potassium citrate (5 mL/L) and HA (2 g/L) + potassium citrate (10 mL/L) showed the presence of allitridin in percentages of 36.02 and 37.69%, respectively.

Methyl allyl disulfide was found in the extracted EOs in the range of 7.85% (plants treated with HA (1 g/L) + potassium citrate (10 mL/L) and 10.06% (plants treated with HA (0 g/L) + potassium citrate (5 mL/L), followed by 10.03, 9.93, and 9.91% as untreated plants, treated with HA (2 g/L) + potassium citrate (10 mL/L), and with HA (2 g/L) + potassium citrate (5 mL/L), respectively.

The highest percentages 13.48%, 13.06%, and 13.01%, of allyl tetrasulfide were found in the EOs extracted from untreated plants, treated with HA (2 g/L) + potassium citrate (5 mL/L), and with HA (2 g/L) + potassium citrate (10 mL/L), respectively, while the lowest value (9.85%) was found in the EO from plants treated with HA (1 g/L) + potassium citrate (10 mL/L).

Other organosulfur compounds like (*E*)-1-allyl-2-(prop-1-en-1-yl)disulfane, (*Z*)-1-Allyl-2-(prop-1-en-1-yl)disulfane, dipropyl disulfide, 1-allyl-3-(2-(allylthio)propyl)trisulfane, 2-[bis(methylsulfanyl)methylene]cyclohexanone and 1-allyl-3-(2-(allyldisulfanyl)propyl)trisulfane were found with minor percentages in all the extracted EOs.

### Antifungal activity of garlic essential oils

The effectiveness of EO from garlic bulbs of plants treated with various concentrations of HA (g/L) + potassium citrate (mL/L) against the growth of *Macrophomina phaseolina* (AUMC15578) and *Fusarium proliferatum* has been investigated. The outcomes demonstrated that *F. proliferatum* and *M. phaseolina* growth were significantly impacted by gradually increasing the concentration of garlic EO used. For instance, the garlic EO at a concentration of 4000 mg/L from plants treated with HA (g/L) + potassium citrate (mg/L) at levels of (2 g/L + 5 mL/L and 2 g/L + 10 mL/L), where the highest percentage of fungal growth *M. phaseolina* was (87.22% and 88.89), respectively (Table 3 and Fig. S2), and the highest Inhibition percentage was (89.79% and 89.89%) against the growth of *F. proliferatum* in the same of concentration (Table 3 and Fig. S3).

**Table 3:** Inhibition percentages of fungal growth (%) of *Macrophomina phaseolina* with essential oils extracted from garlic cloves treated with humic acid (g/L) + potassium citrate (mL/L) treatments

Humic acid (g/L) + Potassium citrate (mL/L)	Garlic EO concentrations (µg/mL)	*Macrophomina phaseolina*		*Fusarium proliferatum*	
		Inhibition percentage	MIC (µg/mL)	Inhibition percentage	MIC (µg/mL)
0 + 0	Control	0.00m	60	0.00n	250
	4000	75.00 ± 1.29c-e		80.00 ± 1.22b	
	1000	30.55 ± 1.27jk		17.25 ± 1.27i	
	250	28.33 ± 1.27k		5.00 ± 1.27j-l	
	60	17.22 ± 1.23l		1.11 ± 1.25l-n	
	15	0.00m		0.00n	
0 + 5	4000	76.11 ± 1.28cd	60	81.33 ± 1.23ab	250
	1000	39.44 ± 1.28hi		20.55 ± 1.27hi	
	250	28.33 ± 1.27k		5.25 ± 1.27j-l	
	60	18.33 ± 1.24l		1.66 ± 1.26l-n	
	15	0.00m		0.00n	
0 + 10	4000	76.11 ± 1.28cd	60	84.89 ± 1.26a	250
	1000	51.66 ± 1.29g		29.44 ± 1.27ef	
	250	31.66 ± 1.26jk		9.44 ± 1.25j	
	60	18.33 ± 1.24l		2.22 ± 1.24k-n	
	15	0.00m		0.00n	
1 + 0	4000	76.11 ± 1.28cd	60	85.33 ± 1.26a	250
	1000	62.77 ± 1.28f		39.44 ± 1.28d	
	250	35.00 ± 1.28ij		9.44 ± 1.27j	
	60	21.66 ± 1.28l		2.77 ± 1.28k-n	
	15	0.00m		0.27 ± 1.23n	
1 + 5	4000	77.22 ± 1.28c	60	85.89 ± 1.27a	250
	1000	70.55 ± 1.28e		40.55 ± 1.26d	
	250	40.55 ± 1.28h		9.44 ± 1.26j	
	60	28.33 ± 1.26k		3.88 ± 1.26k-m	
	15	0.00m		0.55 ± 1.21mn	
1 + 10	4000	82.77 ± 1.29b	60	87.68 ± 1.25a	250
	1000	70.55 ± 1.28e		41.66 ± 1.28d	
	250	41.66 ± 1.26h		19.11 ± 1.27i	
	60	29.44 ± 1.26k		7.22 ± 1.24jk	
	15	0.00m		0.55 ± 1.21mn	
2 + 0	4000	86.22 ± 1.29ab	60	87.89 ± 1.25a	250
	1000	72.22 ± 1.28de		51.66 ± 1.26e	
	250	42.77 ± 1.28h		25.00 ± 1.26f-h	
	60	31.66 1.28jk		9.44 ± 1.25j	
	15	0.00m		0.55 ± 1.22mn	
2 + 5	4000	87.22 ± 1.28ab	60	89.79 ± 1.28a	250
	1000	73.88 ± 1.28c-e		70.88 ± 1.28b	
	250	61.66 ± 1.28F		28.33 ± 1.28e-g	
	60	42.77 ± 1.28h		23.77 ± 1.28gh	
	15	0.00m		0.55 ± 1.21mn	
2 + 10	4000	88.89 ± 1.28a	60	89.89 ± 1.29a	250
	1000	82.77 ± 1.29		71.55 ± 1.29b	
	250	71.66 ± 1.25de		38.33 ± 1.26d	
	60	49.44 ± 1.27g		32.77 ± 1.26e	
	15	0.00m		1.38 ± 1.22l-n	
L.S.D 0.05		4.89		4.71	

Means with the same letter/s with the same column are not significant according to LSD at 0.05 level of probability

As shown in Table 3, the MIC value against the growth of *Macrophomina phaseolina* was 250 mg/L for all EOs extracted from garlic plants treated with various concentrations of HA acid + potassium citrate. Whereas it was 60 mg/L for EOs extracted from garlic plants treated with HA (g/L) + potassium citrate (mL/L) at 2 + 5 and 2 + 10 and reached 250 mg/L for other treatments.

## Discussion

Depending on the dosage of humic acid (HA) and potassium citrate employed, the productivity and bulb diameter of garlic, the bioactive components in terms of total phenol content, antioxidant activity, total carbohydrate, and Sulphur content were greatly improved. The productivity and bioactive components of garlic are highly influenced by many agricultural and environmental conditions, including fertilization. Previous research has confirmed the beneficial effects of HA with potassium fertilization on some vegetable yields (Ibrahim et al. 2015, 2020; Mohammed and Ibraheem 2020; Mohammed et al. 2021). According to some reports, humic compounds are primarily employed to mitigate the detrimental effects of chemical fertilizers on the soil and have a significant impact on overall yield (Shafeek et al. 2015; Badawy et al. 2019). Additionally, HA promotes plant growth by assimilating major and minor nutrients, activating or inhibiting enzymes, altering membrane permeability, promoting protein synthesis, and ultimately triggering the generation of biomass (Ampong et al. 2022). In comparison to other treatments, the improved productivity at larger applications of HA with potassium citrate was likely caused by the availability of more nutrients, which promoted more vegetative development and yield (Ahmed et al. 2010). When compared to the control treatment, foliar application of a liquid humic extract from vermicompost at a ratio of 1:40 v: v improved the productive, commercial, and internal quality characteristics of garlic fruits (Balmori et al. 2019). Ammonium sulfate treatment at the rate of 150 kg/ha and HA at 3000 mg/L increasing yield and yield components of garlic except the clove weight (Zeinali and Moradi 2015).

K is known to have a substantial impact on plant growth. This effect may be related to its relationship with the effectiveness of leaves assimilating CO_2_, activating phytohormones, regulating cellular pH, improving nitrogen uptake, and acting as an activator to enzymatic systems (El-Beltagi et al. 2017). The beneficial effects of potassium citrate can be attributed to citric acid’s role in respiration pathways and the production of crucial energy (ATP synthesis) for all essential cell processes, or to potassium’s roles in photosynthesis and osmoregulation, which enable the import of nutrients from sources to fruits and increase fruit weight (Okba et al. 2021). The increase in bulb diameter and productivity may be a result of how it affects a plant’s K function and increases yield. In addition to being needed for growth, K also keeps cells healthy and controls plant water content (Hafez et al. 2019). The use of foliar K fertilizer has sped up the synthesis of stored carbohydrates that came about as a result of an increase in bulb size as measured by diameter and, eventually, yield. Similar results with garlic (Talware et al. 2012) and onion (Mamdouh et al. 2017) were also seen. As a foliar spray and a soil application, potassium humate, molybdenum, and halex-2 treatments on garlic plants increased plant height, leaf count, neck and bulb diameter, total dry weight per plant, N, P, and K contents in bulbs and leaves, as well as overall yield/fed (Mohsen et al. 2017).

Yields of Garlic EOs from bulbs harvested from plants that received a variety of HA and potassium citrate treatments ranged from 0.34 to 1.41%. Previous works showed that pale yellow EOs were produced using three distinct methods with yields of 0.2%, 0.22%, and 0.18% of Spanish garlic (Satyal et al. 2017). The yield obtained in essential oils from Moroccan garlic was 0.32% (fatima Douiri et al. 2013), 0.72% from Algerian garlic (Boukeria et al. 2016), 0.6 and 0.5% (w/w) for freeze- and oven-dried samples, respectively from Tunisian garlic (Dziri et al. 2014) and the yield of Italian garlic was 0.25% (Casella et al. 2013).

Garlic is known as a S-demanding crop as it is a component of secondary compounds that not only control the taste and medicinal properties of garlic but also are important for resistance against pests and diseases. The highest concentration of Sulphur was observed in bulbs of plants treated with HA (2 g/L) + potassium citrate (5 mL/L), and a similar result was also reported by Jiku et al. (2020). Several Organic Sulphur Compounds (OSCs) such as dimethyl trisulfide, diallyl disulfide, allitridin, allyl tetrasulfide, dipropyl disulfide, methyl 2-propenyl trisulfide, and methyl allyl disulfide were identified in the EOs from garlic bulbs according to the variety of treatments in the present work. Diallyl sulfide, diallyl disulfide (garlicin), diallyl trisulfide (allitridin), andajoene, and vinyl-dithiins are among the OSCs that can be formed in vitro from the unstable allicin (Harris et al. 2001; Amagase 2006).

Diallyl disulfide was found in percentages of 20.51, 25.11, 13.59, and 16.22% in the volatile oils from garlic Balady, EGA 4, EGA 3, and Growers clone, respectively (Anwar et al. 2018). Diallyl trisulfide and diallyl tetrasulfide, the principal components of strong garlic odor, were identified in the distilled EO (Kimbaris et al. 2006; Gong et al. 2021). When the nitrogen fertilizer rates increased, a higher content of alliin was found (Higuchi et al. 2003). The foliar application of Se (10 µg/mL) and HA (10 kg/ha) with irrigation with water at low rates increased the antioxidant activity but negatively affected the allicin content (Ghasemi et al. 2015). Allicin, can hydrolyze giving diallyl disulfide and trisulfide (Yi and Su 2013; Majewski 2014; Salehuddin et al. 2021). Diallyl trisulfide (Allitridin), is one of the most potent of several produced by the hydrolysis of allicin (Block 2010; Cardellina 2013). Crushing garlic can result in the production of diallyl disulfide, which is a byproduct of allicin’s decomposition (Cheng et al. 2016). A high percentage of trisulfide, di-2-propenyl (46.52%) was found in Moroccan garlic EO followed by disulfide, di-2-propenyl, and trisulfide, methyl 2-propenyl (fatima Douiri et al. 2013). Another study observed that diallyl disulfide (25.2%), allyl methyl trisulfide (23.8%), and diallyl trisulfide (21.1%) were the main compounds in the Egyptian garlic EO (Romeilah et al. 2010). Additionally, components of diallyl trisulfide (allitridin) (33.4%), diallyl disulfide (20.8%), and allyl methyl trisulfide (19.2%) were identified as the main compound of the EO of garlic (Satyal et al. 2017). The EOs had a high concentration of sulfur compounds (84.3–98.9%), with diallyl trisulfide (37.3–45.9%), diallyl disulfide (17.5–35.6%), and methyl allyl trisulfide (7.7–10.4%) being the main constituents (Dziri et al. 2014).

*Fusarium proliferatum* and *Macrophomina phaseolina* colony growth were completely suppressed by garlic EOs. Another study claimed that the main chemical components of garlic EO were dimethyl trisulfide, diallyl disulfide, and methyl 2-propenyl trisulfide (Wang et al. 2019) with antifungal activity against *Phytophthora nicotianae*. Strong fungistatic effects were seen with allyl tetrasulfide and methyl allyl disulfide against several phytopathogenic fungi, including *Fusarium oxysporum*, *Botrytis cinerea*, *Verticillium dahlia*, and *Phytophthora capsici* (Hayat et al. 2016). Garlic EO showed strong antimicrobial activity against the growth of *Staphylococcus aureus*, *Salmomella enteritidis*, *Aspergillus niger*, *Penicillium cyclopium*, and *Fusarium oxysporum* (Benkeblia 2004). Garlic EO can first penetrate the cellular membrane and wall of *Penicillium funiculosum* hyphae before causing membrane detachments that lead to the release of certain macromolecules and cytoplasm. Garlic EO killed *P. funiculosum* cells at various places and eventually caused their lysis (Li et al. 2014). Garlic EO’s primary component, diallyl disulfide, was proposed to have a significant role in the antifungal activity against *Phytophthora nicotianae* by damaging the integrity of the mycelial cell membrane, increasing cell membrane permeability, and inducing cell death (Wang et al. 2019).

It is clear from the data that fertilizing with foliar potassium citrate and applying humic acid to the soil encourages garlic plants to produce more bioactive bulbs and clove oil yield. The best treatments, combining a reasonably high concentration of humic acid with a high or moderate concentration of potassium citrate (5 or 10 mL/L), are highly suggested to increase productivity, yields of essential oils, and bioactive component production. Additionally, essential oils are highly suggested to protect geranium plants against the growth of *Macrophomina phaseolina* and *Fusarium proliferatum*, the two most common root rot and wilt diseases.

The study offers important new information about the effects of some biostimulants on the productivity and bioactive compounds in garlic plants. Furthermore, the essential oils exhibit antifungal properties. However, the results might be strengthened by using a larger range of concentrations, a more comprehensive approach to extraction techniques for other bioactive compounds, and the addition of more fungal isolates. Furthermore, investigating certain antifungal pathways against food-poisonous pathogens and carrying out further evaluations on other species might increase the study’s applicability in the future.

## Electronic supplementary material

Below is the link to the electronic supplementary material.

Supplementary Material 1

## Data Availability

All data generated or analyzed during this study are included in this published article.
