# Supplementary material for: Biostimulants for enhancing productivity, bioactive components, and the essential oils of garlic with the potential antifungal activity
Source: AMB Express. 2024 Nov 27;14:130. doi: 10.1186/s13568-024-01790-5 (PMC11602910; doi:10.1186/s13568-024-01790-5)
Supplement: Supplementary file 1 — Supplementary Material 1 [file 13568_2024_1790_MOESM1_ESM.docx]

Biostimulants for enhancing productivity, bioactive components, and the essential oils of garlic with the potential antifungal activity

Hanaa S. Hassan^1^, Mostafa N. Feleafel^1^, Mina S. R. Abd El-Lahot^2^, Mervat EL-Hefny^3,^*, Taghreed F. M. Abdel Rahman^4^, Abeer A. Mohamed^5^, Doaa Y. Abd-Elkader^1^, R. M. Mahdy^6^

^1^ Department of Vegetable, Faculty of Agriculture (El-Shatby), Alexandria University, Alexandria 21545, Egypt

^2^ Department of Food Science & Technology, Faculty of Agriculture (El-Shatby), Alexandria University, Alexandria 21545, Egypt

^3^ Department of Floriculture, Ornamental Horticulture and Garden Design, Faculty of Agriculture (El-Shatby), Alexandria University, Alexandria 21545, Egypt

^4^ Department of Ornamental, Medicinal and Aromatic Plant Diseases, Plant Pathology Research Institute, Agricultural Research Center (ARC), Giza 12619, Egypt

^5^ Plant Pathology Institute, Agricultural Research Center (ARC), Alexandria 21616, Egypt

^6^ Horticulture Department, Faculty of Agriculture, Tanta University, Tanta 31527, Egypt

*** Corresponding author:** [mervat.mohamed@alexu.edu.eg](mailto:mervat.mohamed@alexu.edu.eg)

Table S1. Level-adjusting procedures for humic acid and potassium citrate

| Treatment number | Level of Humic acid (g/L) | Level of potassium citrate (mL/L) |
| --- | --- | --- |
| 1 | 0 | 0 |
| 2 | 0 | 5 |
| 3 | 0 | 10 |
| 4 | 1 | 0 |
| 5 | 1 | 5 |
| 6 | 1 | 10 |
| 7 | 2 | 0 |
| 8 | 2 | 5 |
| 9 | 2 | 10 |


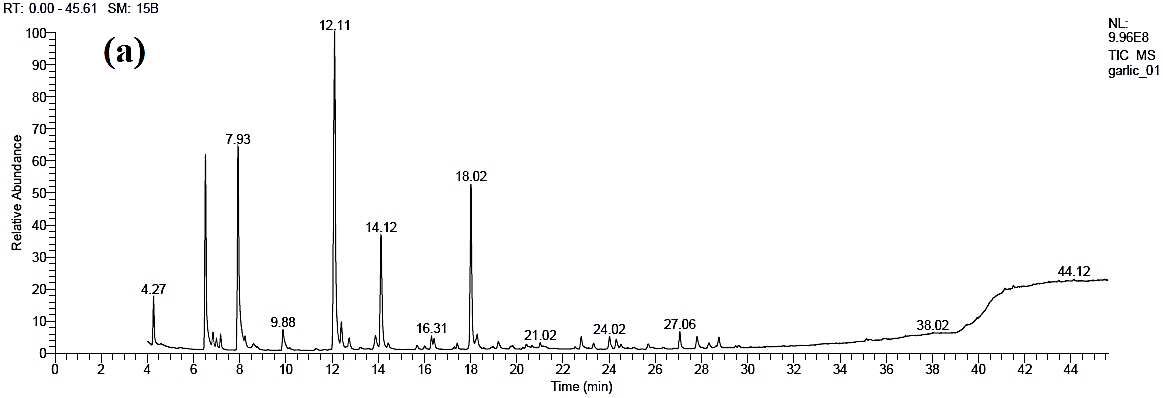


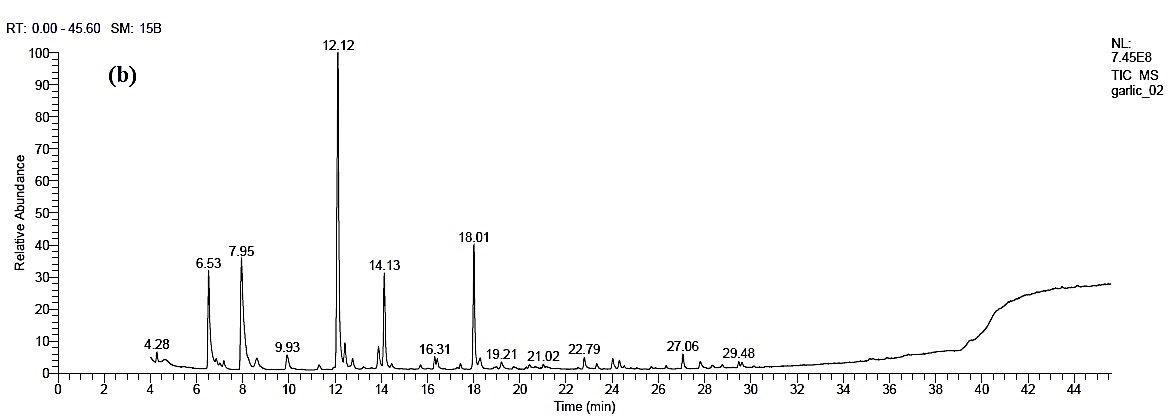


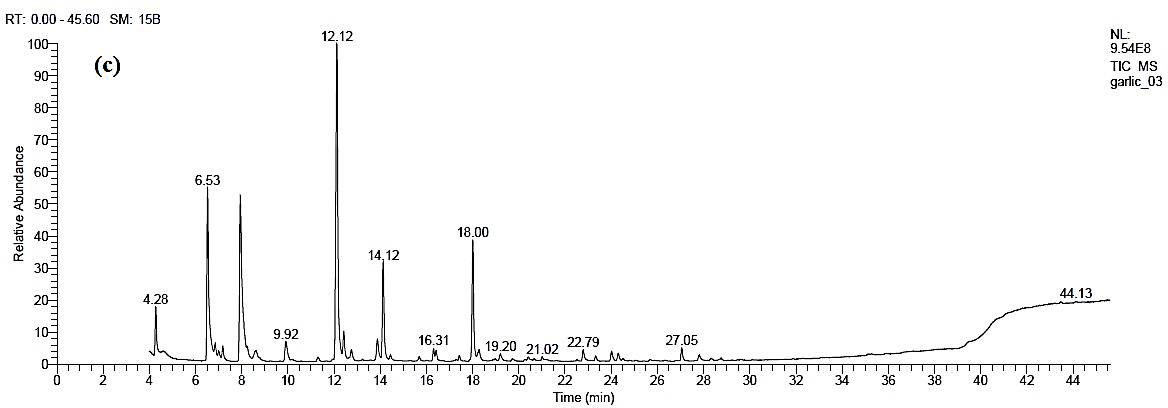


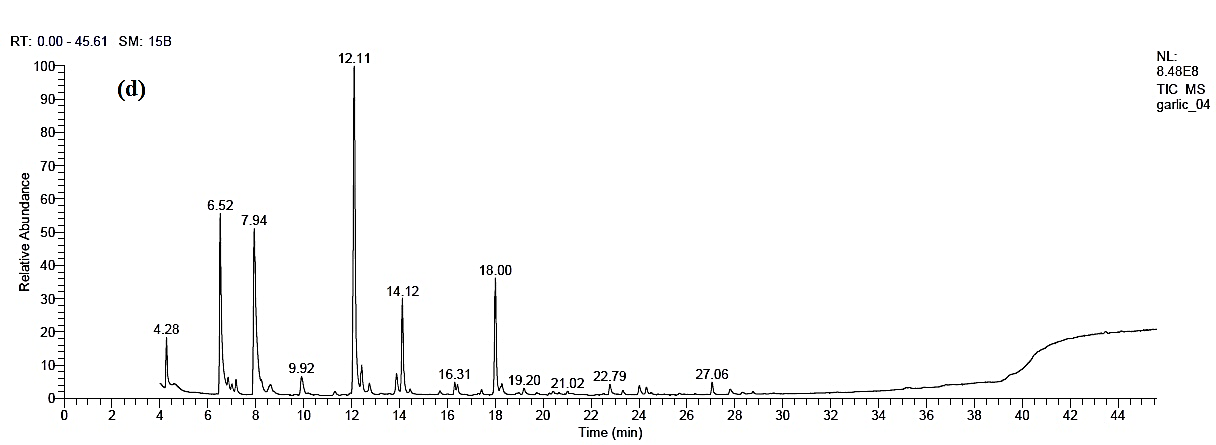


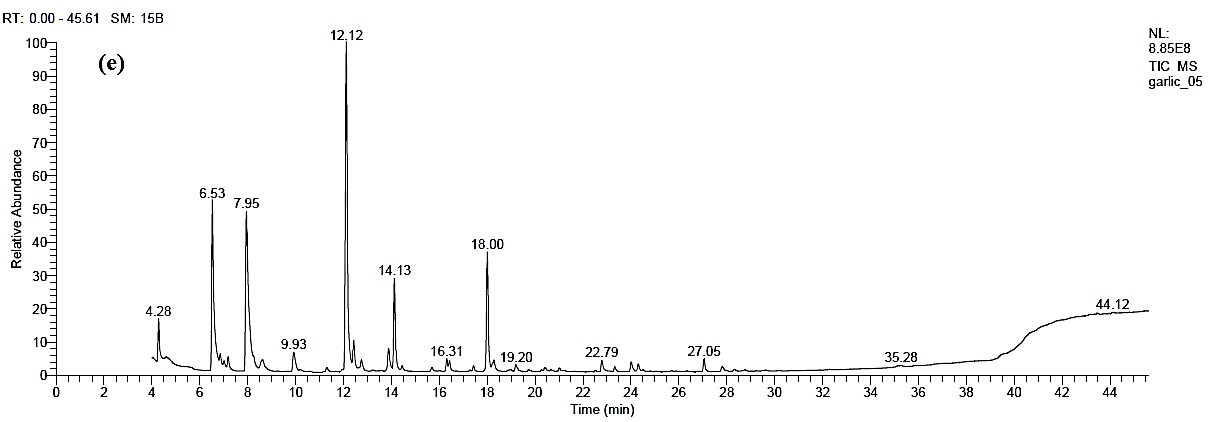


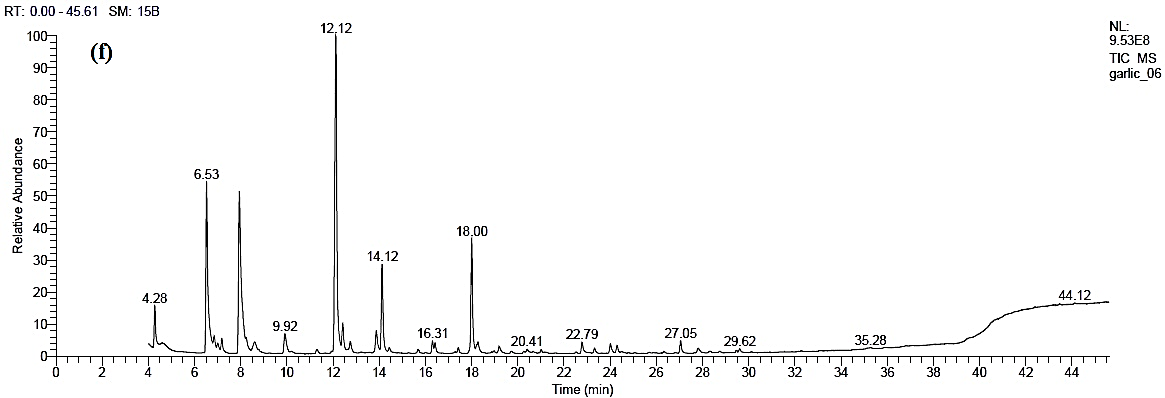


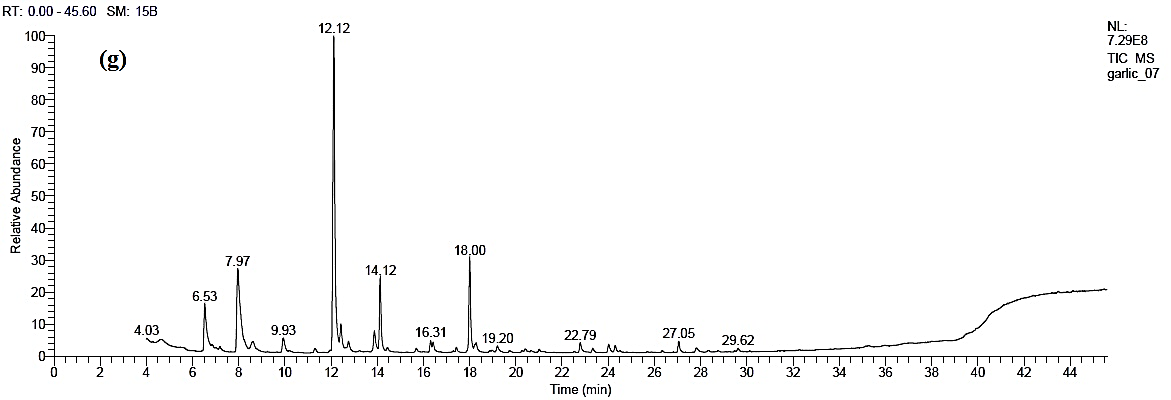


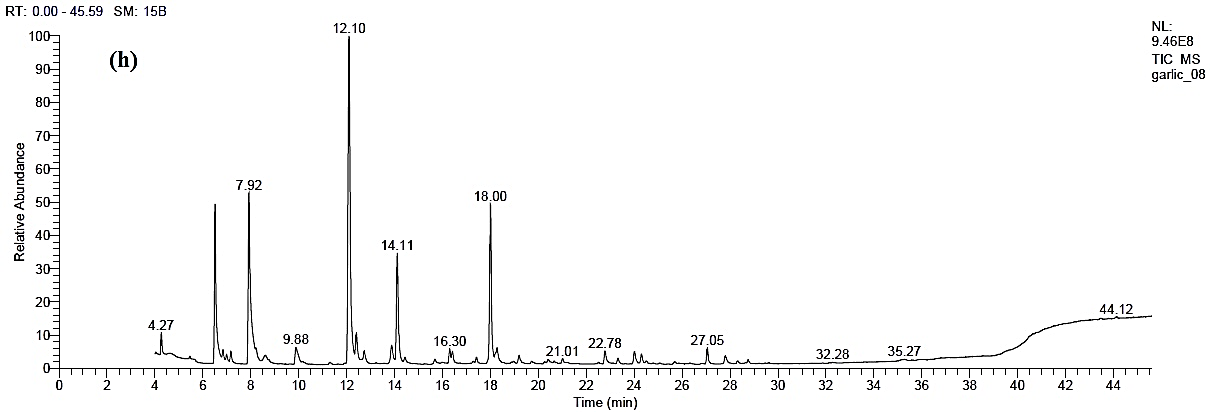


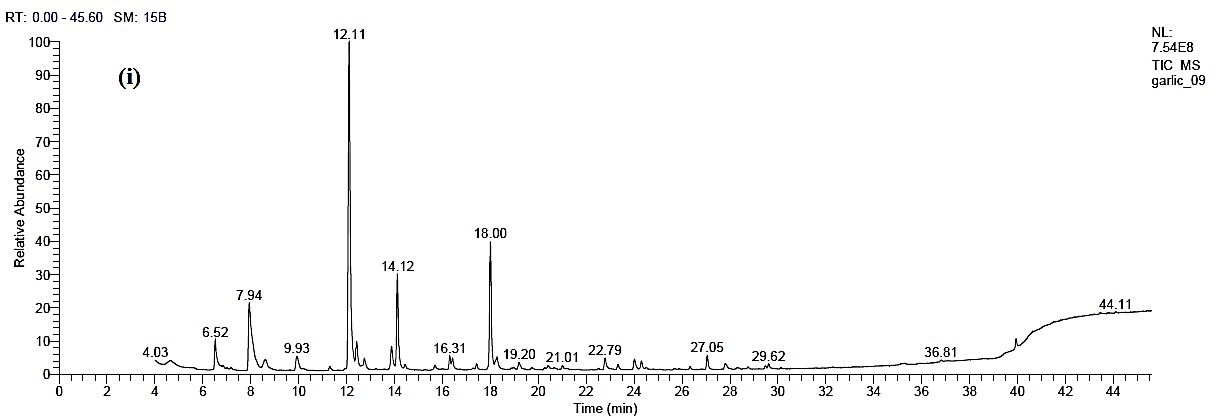


Figure S1. GC-MS chromatograms of the identified chemical compounds in garlic essential oils


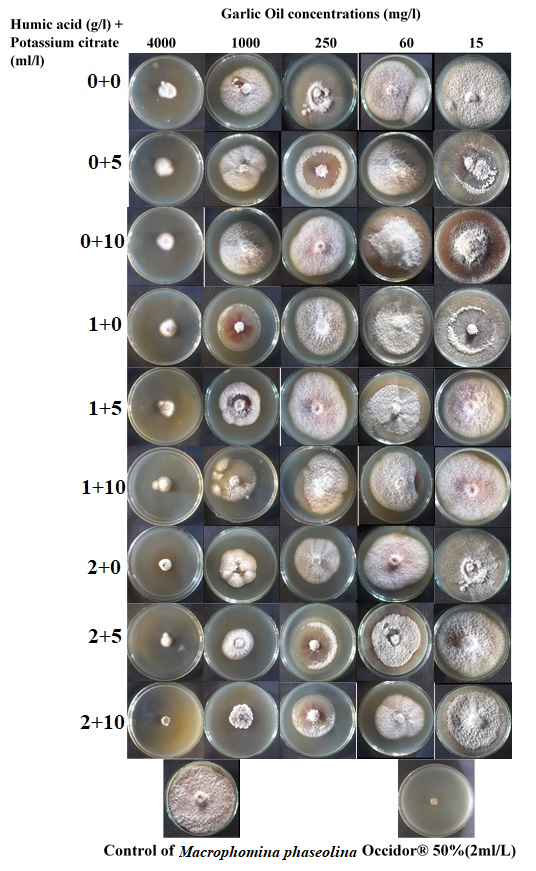


Figure S2. Antifungal bioassay of Garlic EO against the mycelial growth of *Macrophomina phaseolina*

**
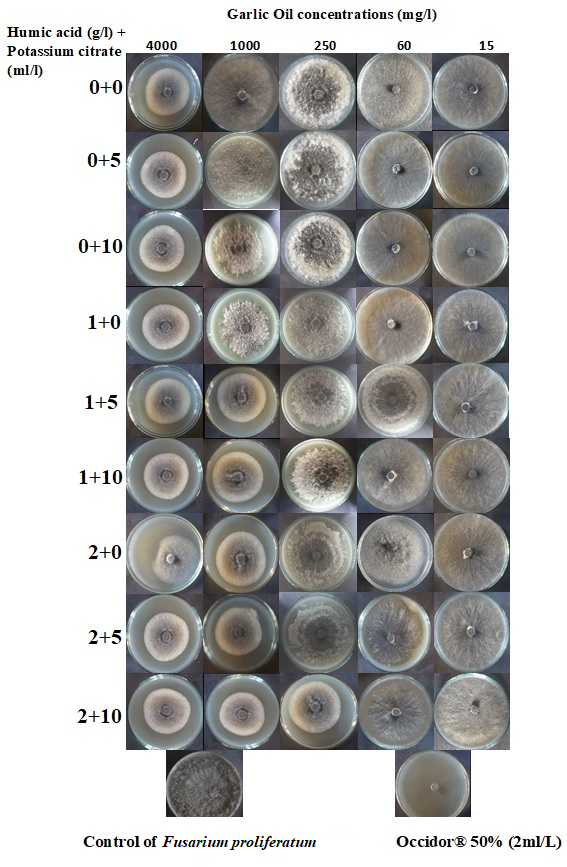
**

Figure S3. Antifungal bioassay of garlic EO against the mycelial growth of *Fusarium proliferatum*
